# Portrait of intense communications within microfluidic neural networks

**DOI:** 10.1038/s41598-023-39477-9

**Published:** 2023-07-29

**Authors:** Victor Dupuit, Anne Briançon-Marjollet, Cécile Delacour

**Affiliations:** 1 Institut Néel, University Grenoble Alpes, CNRS, Grenoble INP, 38000 Grenoble, France; 2 HP2 Laboratory, University Grenoble Alpes, Institut National de la Santé et de la Recherche Médicale U1300, Grenoble, France

**Keywords:** Neural circuits, Techniques and instrumentation, Biomedical engineering, Nanobiotechnology

## Abstract

In vitro model networks could provide cellular models of physiological relevance to reproduce and investigate the basic function of neural circuits on a chip in the laboratory. Several tools and methods have been developed since the past decade to build neural networks on a chip; among them, microfluidic circuits appear to be a highly promising approach. One of the numerous advantages of this approach is that it preserves stable somatic and axonal compartments over time due to physical barriers that prevent the soma from exploring undesired areas and guide neurites along defined pathways. As a result, neuron compartments can be identified and isolated, and their interconnectivity can be modulated to build a topological neural network (NN). Here, we have assessed the extent to which the confinement imposed by the microfluidic environment can impact cell development and shape NN activity. Toward that aim, microelectrode arrays have enabled the monitoring of the short- and mid-term evolution of neuron activation over the culture period at specific locations in organized (microfluidic) and random (control) networks. In particular, we have assessed the spike and burst rate, as well as the correlations between the extracted spike trains over the first stages of maturation. This study enabled us to observe intense neurite communications that would have been weaker and more delayed within random networks; the spiking rate, burst and correlations being reinforced over time in terms of number and amplitude, exceeding the electrophysiological features of standard cultures. Beyond the enhanced detection efficiency that was expected from the microfluidic channels, the confinement of cells seems to reinforce neural communications and cell development throughout the network.

## Introduction

In vitro model systems are of primary interest to provide user-defined neural architecture and to study cell organization and processes in the laboratory. For that purpose, several methods have been developed to build physiologically relevant neural networks from planar cell cultures^1–3^ to 3D cell cultures^4^ and organs-on-chips^5–7^. These approaches have succeeded in isolating on a chip specific cell mechanisms that could remain overlooked in vivo.

The first method, which consists of dissociated neuron cultures, presents several advantages. Although the structure of tissues is mostly lost, a high degree of biochemical and biophysical control is achievable, in concomitance with miniaturized, highly efficient coupling with electrical devices for long-term recordings. A limitation of such models is the random organization of somas and neurites. This complicates the observation of the same cells or neurites throughout their development and prevents the plasticity of the network from being evaluated. Additionally, different populations cannot be separated, which restricts the study to intrapopulation communication only. Thus, several studies have attempted to structure neuronal networks in vitro by confining soma location and neurite outgrowth, providing a suitable way to study individual neurons and their interaction over the course of weeks. First, the combination of adhesive and repellent polymers enabled the guidance of neurons along defined patterns and led to the first functional architectures built in the laboratory^8–10^. While this approach succeeded in isolating and connecting small numbers of cells, challenges remain in structuring large populations. Additionally, neurons can still reach undesired areas after a couple of weeks. Thus, microstructures were developed to provide additional physical barriers and to prevent motile cells from migrating^11^. Among them, PDMS-based microfluidic circuits have emerged as highly versatile tools providing many suitable properties for positioning, culturing and interfacing large populations of neurons^12,13^. Since the first demonstrations, PDMS-based microfluidics has been used for modeling brain circuits on a chip^14–17^, as well as for single-neuron analysis^18–22^. This approach combines neuron-adhesive coating and physical barriers for efficient cell adhesion and time-stable architectures^13,23–26^ while maintaining high optical transparency for high-resolution imaging^27,28^. Additionally, microfluidic devices can be assembled with any substrate, including electrical device arrays^4,29–34^ for monitoring the activity of the same cell and how its activity evolves over time. Indeed, this combination fulfils essential conditions, which are long-term maintenance of the defined neural circuit^35,36^ and efficient neuron-device coupling^37–40^.

**Figure 1:**
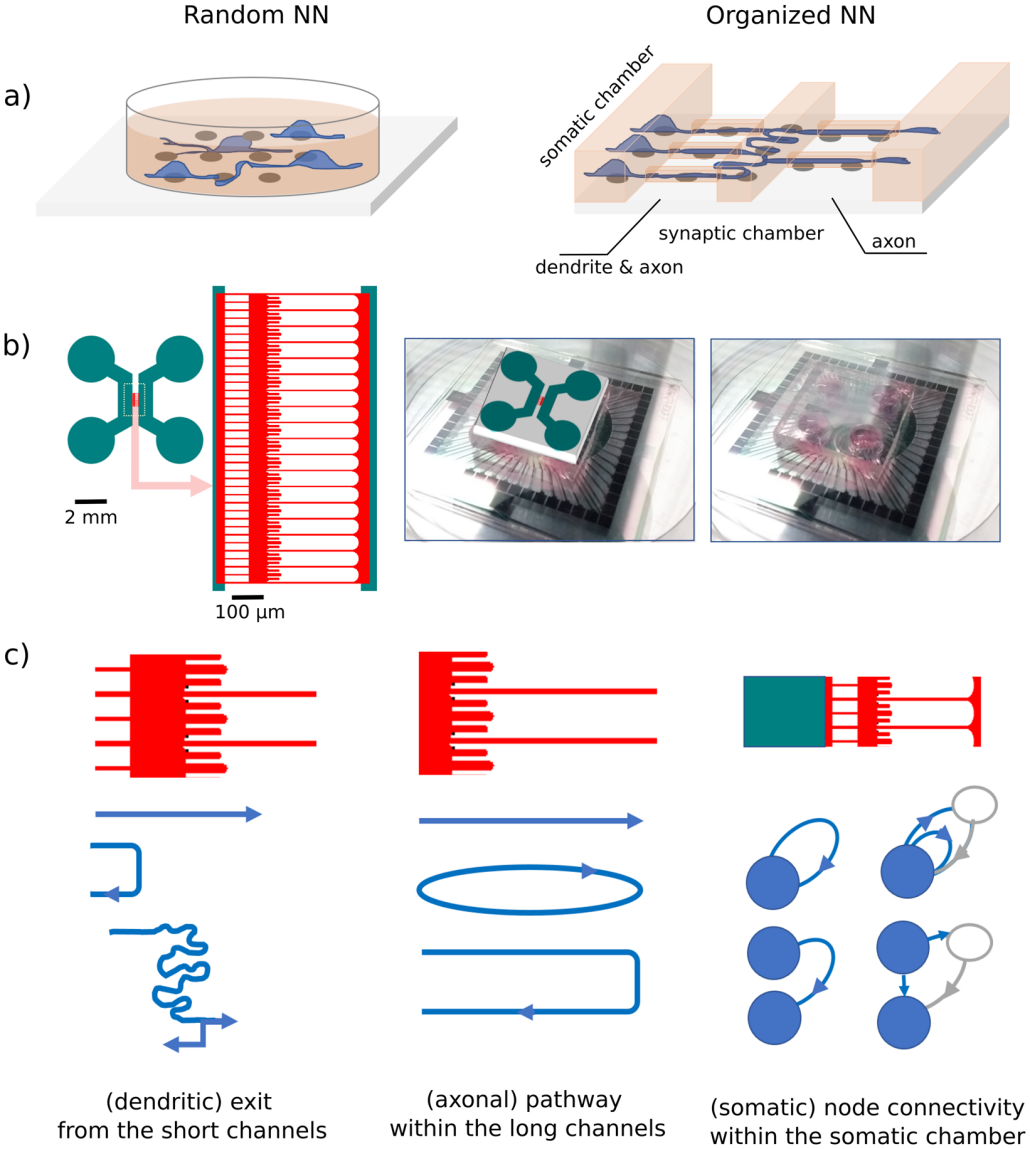
Description of the neural networks (NNs) (**a**) showing the expected network architectures within the random (left) and microfluidic (right) samples. (**b**) The layout and optical micrograph of the microfluidic NNs assembled with the microelectrode arrays. The dissociated neurons extracted from mouse embryo hippocampi are seeded within the mm-wide fluidic channels of the microfluidic chip (teal). While soma can attach and explore the somatic chamber only, neurites also show outgrowth within the narrow microchannels and the synaptic chamber (red area). (**c**) Layout of the microfluidic chip used to induce directional communication. For each microfluidic compartment, the schematics illustrate examples of geometrically driven pathways for dendrites and axons and examples of resulting nodes (afferent and efferent somas) that could be expected within the somatic chambers (active and hidden nodes in blue and gray, respectively).

Such hybrid electronic and microfluidic platforms have been widely used, for instance, to study spike propagation^40–47^ and structure‒function relationships^8,48–50^. As an example, the widely used dual-compartment design enables the somas to be kept in large somatic chambers to isolate populations, while the axons and dendrites explore narrow fluidic channels aligned with the sensing devices (Fig. 1). Thus, a neuronal network (NN) with localized and tunable connectivity could be obtained, providing key building blocks for a topological network, i.e. one with geometrically defined functional properties.

On the other hand, microfluidic circuits impose important spatial restrictions on growth compared to the standard culture conditions in open liquid media. These constraints are expected to shape the network organization as well as to impact cell development^24^. It is therefore of primary interest to assess to what extent a microfluidic circuit induces significant changes in the neural communications and electrical maturation of a network. In particular, connectivity is expected to play an important role in modulating NN maturation but often remains hidden or difficult to assess within standard culturing conditions.

Here, we investigated the establishment of electrical activity and the emergence of spatiotemporal correlations within organized (microfluidic) and random (control) neural networks from the same batches of dissociated neurons (Fig. 1a). The defined NNs were cultured on an array of microelectrodes to assess several electrophysiological features in order to investigate the impacts of the growth environment on cell maturation and on the emergence of activity patterns.

## Results and discussion

### Construction of in vitro neural networks (NN)

The topology for the microfluidic NNs was designed as a dual-compartment architecture separated by microchannels and a middle chamber, as described in Fig. 1a and b. The microfluidic design includes large channels (teal area) on both sides of the microfluidic circuit, which are for seeding somas. Physical barriers prevent the somas from migrating outside these large chambers. However, the 5-µm-tall microchannels and a middle chamber (red area) enable neurites to spread and connect the fluidic compartments along defined pathways. Because of the enhanced growth kinetics of the axons, long, straight microchannels (> 500 µm in length) are expected to favor them and to prevent dendrites from connecting distant populations.

Figure 1c illustrates the possible neurite guidance and connection schemes. From left to right, the first and shortest microchannels should favor neurite outgrowth from the somatic to the synaptic chamber. From there, dendrites are expected to spread over this 3-mm-wide middle chamber, while the axons, in contrast, may grow straight ahead toward the opposite channels or turn back toward the somatic chamber. At one entrance of the long axon microchannels, short dead-end microchannels should prevent an axonal closed loop, which would lock axons into the long microchannel. Those traps should guide the axons toward the short microchannel and the somatic chamber. The last schematic illustrates a simple, inexhaustive list of examples of connectivity that may result from these guiding rules in the cases of one or two nodes located in a somatic chamber. Active and hidden nodes (blue and gray circles, respectively) can both be involved.

**Figure 2:**
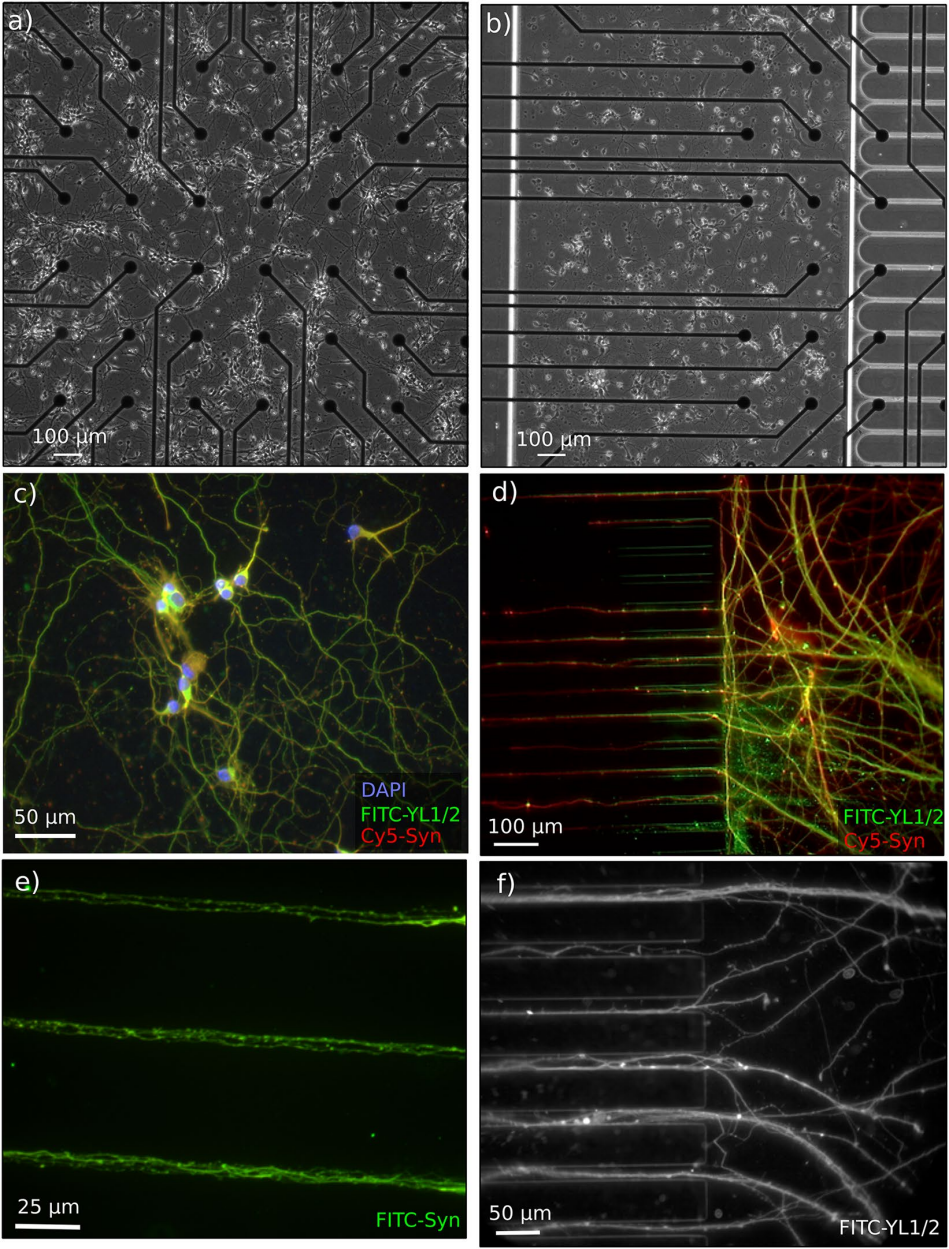
Optical and fluorescent micrographs of random and microfluidic networks showing the homogeneous distribution of somas within the random area of both control (**a**) and microfluidic (**b**,**c**) samples and the wide exploration of neurites within all fluidic compartments, including the somatic chamber (**c**), the microchannels and the synaptic chamber (**d**–**f**). Immunofluorescence staining was performed after 14 days in culture. DAPI, anti-synapsin, and anti-tubulin (YL1/2) were chosen as markers for labeling the cell nuclei, synapses and cytoskeleton, respectively.

The microfluidic circuits are then assembled with electronic chips on which microelectrode arrays are accurately aligned with the fluidic compartments and microchannels (Fig. 2). Thus, several recording devices can efficiently track spike propagation within the neurites while simultaneously monitoring soma activation.

For both growth conditions, primary cells extracted from hippocampal neurons were seeded on poly-l-lysine-coated microelectrode arrays and cultured in glial-conditioned media (same culture for both conditions). Thus, the substrate properties and culture conditions remained the same for the two batches of samples (details in “Materials and methods”). In the somatic chamber, neurons were well dispersed, and neurites homogeneously covered the underlying substrate surface, forming a highly entangled mesh (Fig. 2b). Additionally, the synaptic chamber was widely explored by the neurites (Fig. 2d), confirming their efficient spreading within the short microchannels as well as the efficient filtering of somas (Fig. 2e). Figure 2f gives a closer view of the junction with the synaptic chamber. The intricate entanglement of neurites and their proximity within the microchannels is expected to reinforce the neurite coupling efficiency and the network’s modularity. These first results assessed the healthy and efficient outgrowth of neurons in the microfluidic compartments, which succeeded to provide the expected network structure, mainly by keeping the soma and neurite compartments in the desired location.

### Activity pattern of the built NNs

**Figure 3:**
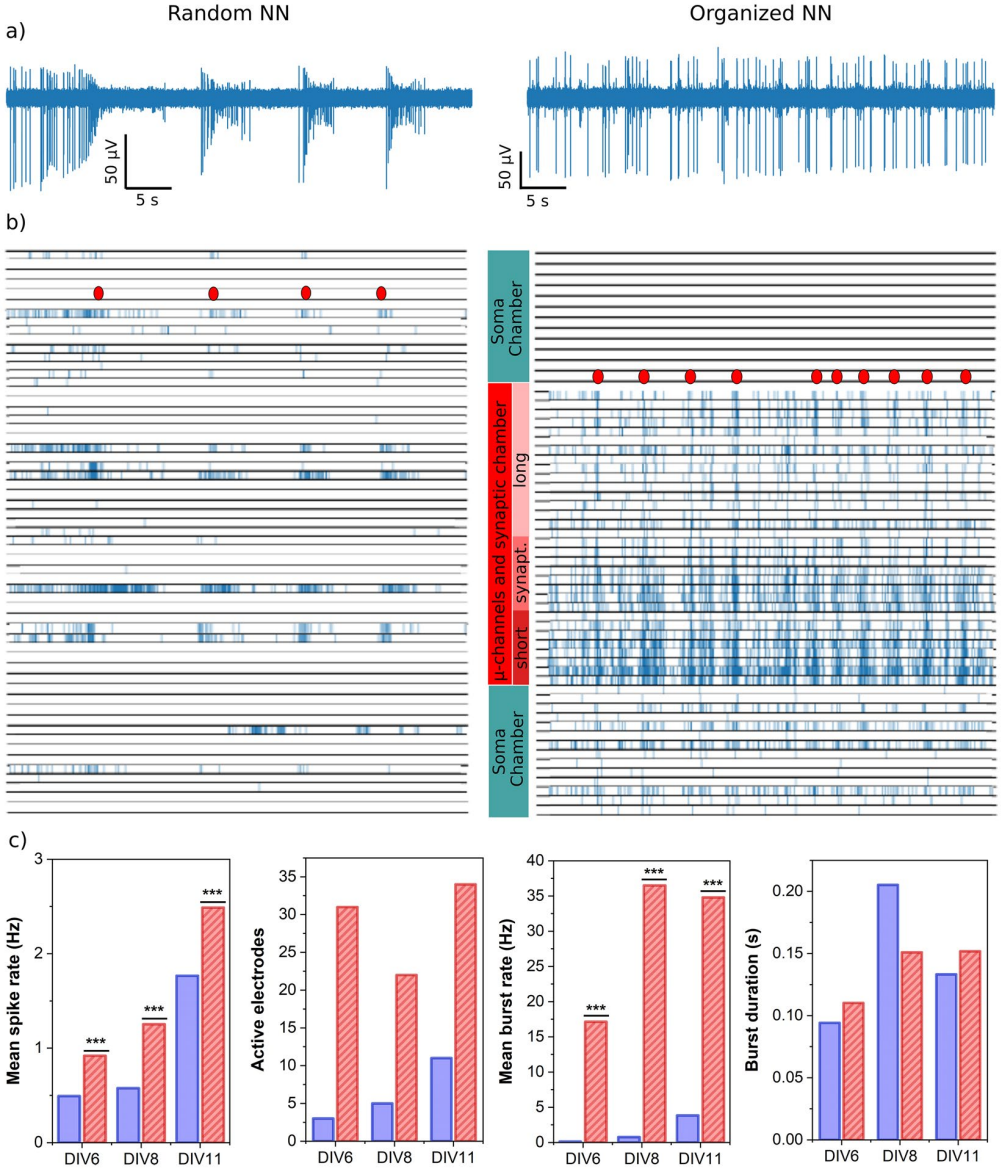
Activity patterns of random and organized NNs. Comparison of the neuronal activity of cultured hippocampal neurons cultured in random configuration (left column) and on a microfluidic chip (right column). Recordings were acquired 6 days after seeding (6 days in vitro). (**a**) Typical 50 s time course of one recording channel of the MEA within the control random sample (left) and inside an axonal microchannel (right). (**b**) Raster plots of all events crossing the negative threshold of 5 mean absolute deviations for the 64 recording channels of the MEA in the control and microfluidic conditions (left and right resp.). Red dots highlight examples of collective bursts. (**c**) Evolution of neural activity during the culture time for random (blue) and organized (red) NNs, in terms of the following (from left to right): mean spike rate per active electrode (min 0.1 Hz mean firing rate), number of active electrodes, mean burst rate and burst duration. The mean spike and burst rates are extracted from the voltage traces for each recording channel and averaged among all active electrodes (60 electrodes total, same culture for all conditions). Statistical significance ***p < 0.001 (Student’s *t* test).

Figure 3 shows the representative activity recorded within the random and organized networks on Day 6 in vitro (DIV6). As clearly observed, the number of active electrodes and the spike rate are significantly higher in the organized microfluidic NN (Fig. 3a and b). Additionally, the number of isolated spikes as opposed to burst events was higher than that in controls (Fig. 3c). Thus, the modularity of microfluidic NNs appears enhanced within the microfluidic network (dual-compartment configuration shown in Fig. S1).

Note that the electrodes located within the microchannels are expected to have a high sealing resistance because the channel cross section is small and filled with cellular material. As a result, the detection efficiency of such electrodes is believed to be increased compared to that of their synaptic and somatic chamber counterparts^44^. This effect related only to the measurement condition could artificially increase the activity level observed in microfluidic NNs. However, the spiking rate measured in the synaptic chamber did not follow that trend. While this compartment was similar to the somatic chamber in terms of growth conditions, the spiking rate was significantly higher, being rather comparable to that of the microchannels. Thus, the recording conditions could not explain the higher electrical activity. The electrical activity was enhanced independently of the MEA’s detection efficiency, revealing the impact of the NN structure on the cell activity and the discrepancy in the spiking dynamics of the soma and the neurite.

The mid-term evolution of the electrical activity remained the same for both conditions, with all electrophysiological features globally increasing over time up to Day 15 (Fig. 3c). Interestingly, the maximal number of active electrodes was reached earlier for the confined microfluidic NN (i.e. 4 days earlier than for the open NN, Fig. S2). Additionally, the number of active electrodes was significantly higher, in agreement with the raster plots (Fig. 3b). Thus, more electrodes were active, and their activation occurred earlier in cell development. The confinement and geometrical constraints of the microfluidic environment reinforce the establishment of electrical activity, which agrees with the accelerated maturation of neuronal cells previously observed by immunohistochemistry within a similar microfluidic chip^24^.

The evolution of the burst rate followed a similar trend, increasing up to Day 14. Values ranged from 24 to 34 Hz for the microfluidic networks, greatly exceeding the bursting rate of random NN (10 times higher). The burst duration was, however, similar for control and microfluidic networks, slightly increasing with the culture time (from 50 to 250 ms) and as expected for hippocampal neurons^4^, confirming the reliability of the microfluidic NNs.

### Spiking dynamics at the cell level

**Figure 4:**
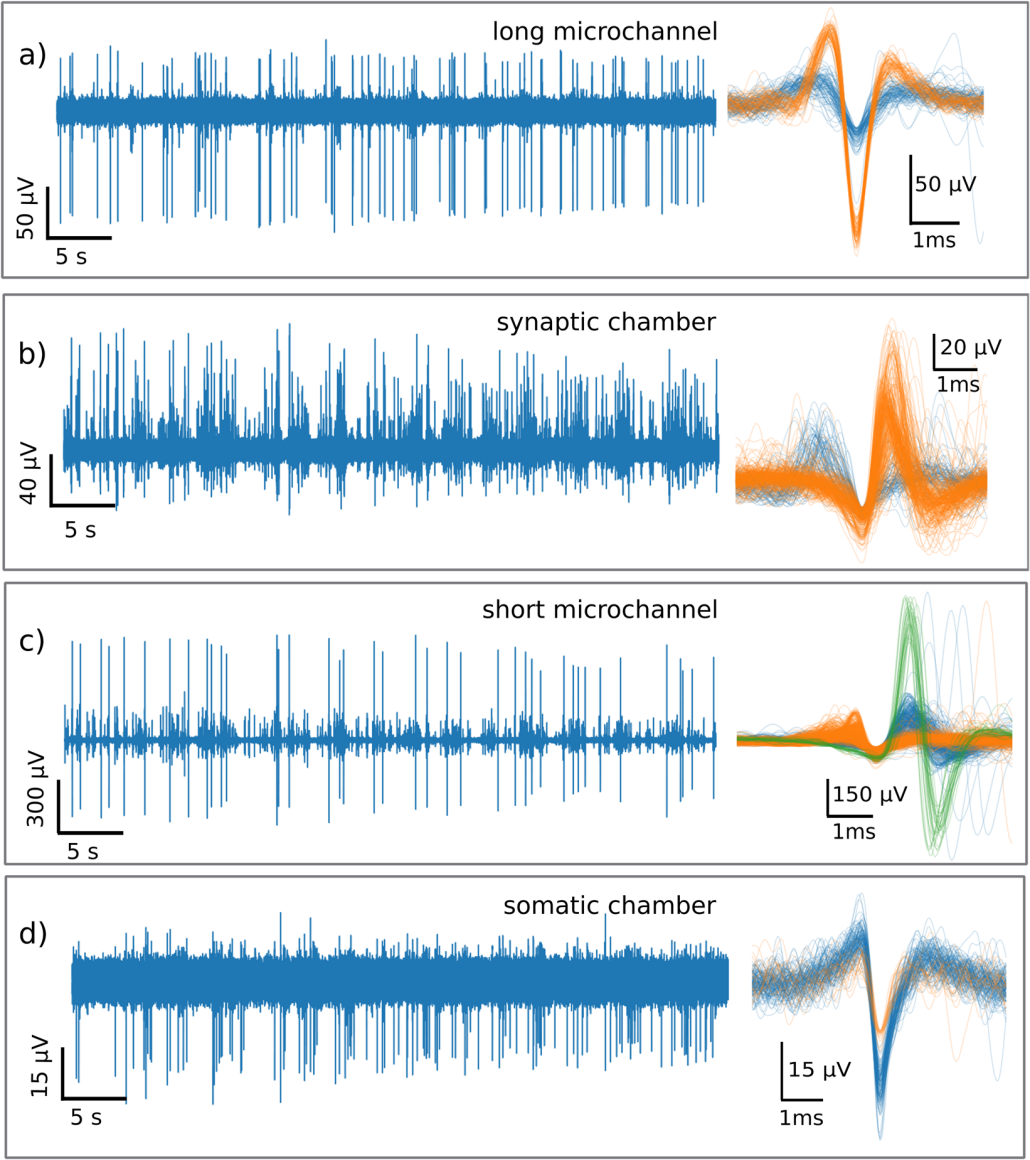
Spike forms acquired in each microfluidic compartment. Data are sourced from the same recording at DIV 11, with the 50 s time trace on the left and the superposed cutouts extracted by a spike sorting algorithm (detailed in methods). From top to bottom, the figure shows the typical voltage time trace and spike forms within the long and distant axonal microchannel; the synaptic middle chamber (without somas); the short neurite (dendrites and axons) microchannels; and the somatic chamber.

Neurite compartments exhibited dense activity patterns compared to the somatic chamber, with the highest spiking rates being located within the proximal compartments that were the closest to the somatic chamber (Fig. 4). Within these short microchannels, spike patterns were characterized by the highest spike amplitude and shape variability. This variability remained within the synaptic chamber, but spike amplitudes were lowered. In those short and synaptic compartments, both dendrites and axons can be expected. However, in the distal and long microchannels, spike amplitude and shape were almost perfectly constant, which is as expected for action potentials carried by axons. These discrepancies were observed under the same growth conditions, all within the microchannels, and stem from the physiological properties of neurites.

Interestingly, the activity in the somatic chamber resembled that of the control samples in terms of spike shape and spike rate (Fig. 3a). When the activity within the somatic chamber was isolated, the spiking rate closely followed the trend observed in control samples, ranging from 0.9 to 2.5 Hz from 6 to 11 days (Fig. S2), which is a typical value for hippocampal neurons. Thus, the areas containing the soma (within the random and organized NNs, respectively) exhibited comparable spike patterns regardless of the growth condition (opened or confined). Previous works reported similar differences between somatic and axonal spikes (without the microfluidic environment)^42^, which agrees with our observations and further highlights the physiological relevance of the observations. Here, the microchannels provided a unique way to identify and study neurite activity in proximal and distant areas, presumably corresponding to dendrites and axons, respectively.

### Spiking dynamics at the network level

**Figure 5:**
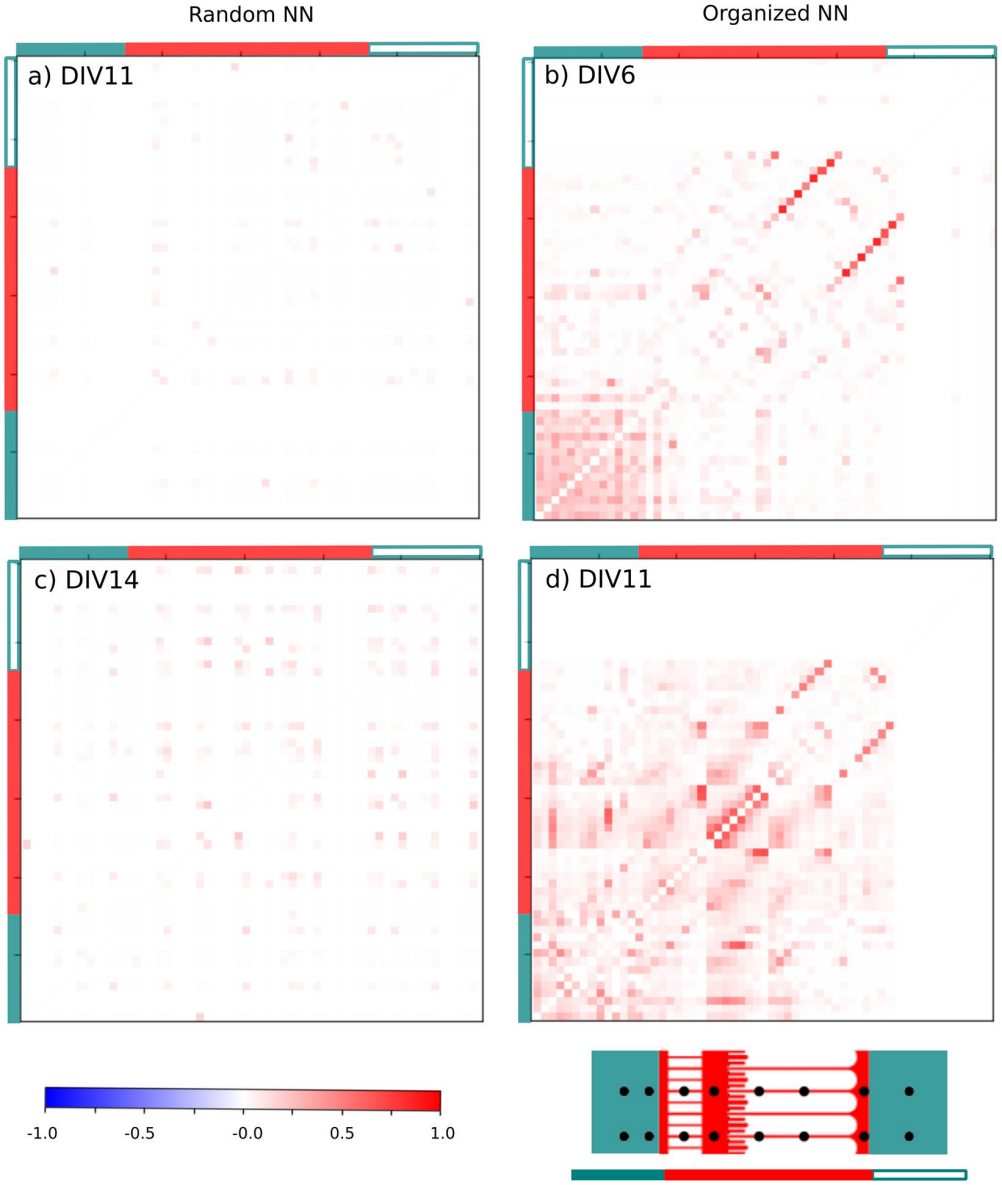
Correlations within random and organized NNs. The cross-correlation matrix (CCM) was extracted from the 60 recording channels of the MEAs during the culture time (one electrode per line and per column; bin size < 5 ms). From top to bottom: CCM obtained at DIV11 and DIV14 for the control sample (left) and at DIV6 and DIV11 for the microfluidic sample (right). (Bottom right) Schematics illustrate the position of the recording channels within the microfluidic compartments. The bottom colored bar is then used in the (x–y) axes of the CC maps to highlight the position of each microelectrode: (filled, teal) in the large chamber containing all the soma, (filled, red) in the microchannels and the synaptic chamber, and (open, teal) in the empty large chamber for axon outputs only (no soma).

The cross-correlation (CC) analysis (Fig. 5) provided a functional cartography of the random and organized networks at several stages of their development (detailed in materials and methods, and see Fig. S3 for the dual-somatic chamber). For the control sample, correlations became significant at DIV11 between electrode clusters randomly dispersed over the whole sample (Fig. 5a). Their amplitude was weak but remained constant over the network. In contrast, cross-correlations were spatially defined and more intense in term of amplitude and number within the organized networks (Fig. 5b), also emerging earlier at DIV5.

Maximal values were found within the long and distal microchannels, with mean correlation coefficients close to 1 and 0.5, respectively. Indeed, strong correlations can be expected when measuring spike propagation within the axonal compartment, which is more highlighted within the distal and long microchannels.

Somatic signals were correlated with some electrodes located in the microchannels and the synaptic chamber, revealing long-range synchrony as well (Fig. 5b). Their amplitudes increased with time (Fig. 5d), revealing a reinforcement of network synchrony and connectivity, especially between the microchannels and the synaptic and somatic chambers. They were concomitant with a modulation of short-range correlations, which became higher between neighboring electrodes. This effect could have several origins, such as time selection of the master node and a reinforcement of selected connections. Additionally, it could result from inhibitory activity, glutamatergic and GABAergic neurons being expected in similar proportions in our culture, and their maturation could explain the appearance of silent electrodes at the final stage of electrical maturation.

Thus, groups of spatially confined electrodes revealed a synchronization of the subpopulation consistent with the geometrical constraints. Somatic and synaptic chambers and neurite microchannels exhibited specific spiking patterns (Figs. 3 and 4) and correlation landscapes (Fig. 5) that enabled the identification of each network compartment. In that way, microfluidic circuits are capable of inducing significant differences in the spatiotemporal dynamics of in vitro neural networks.

### Main paths of neural communication

**Figure 6:**
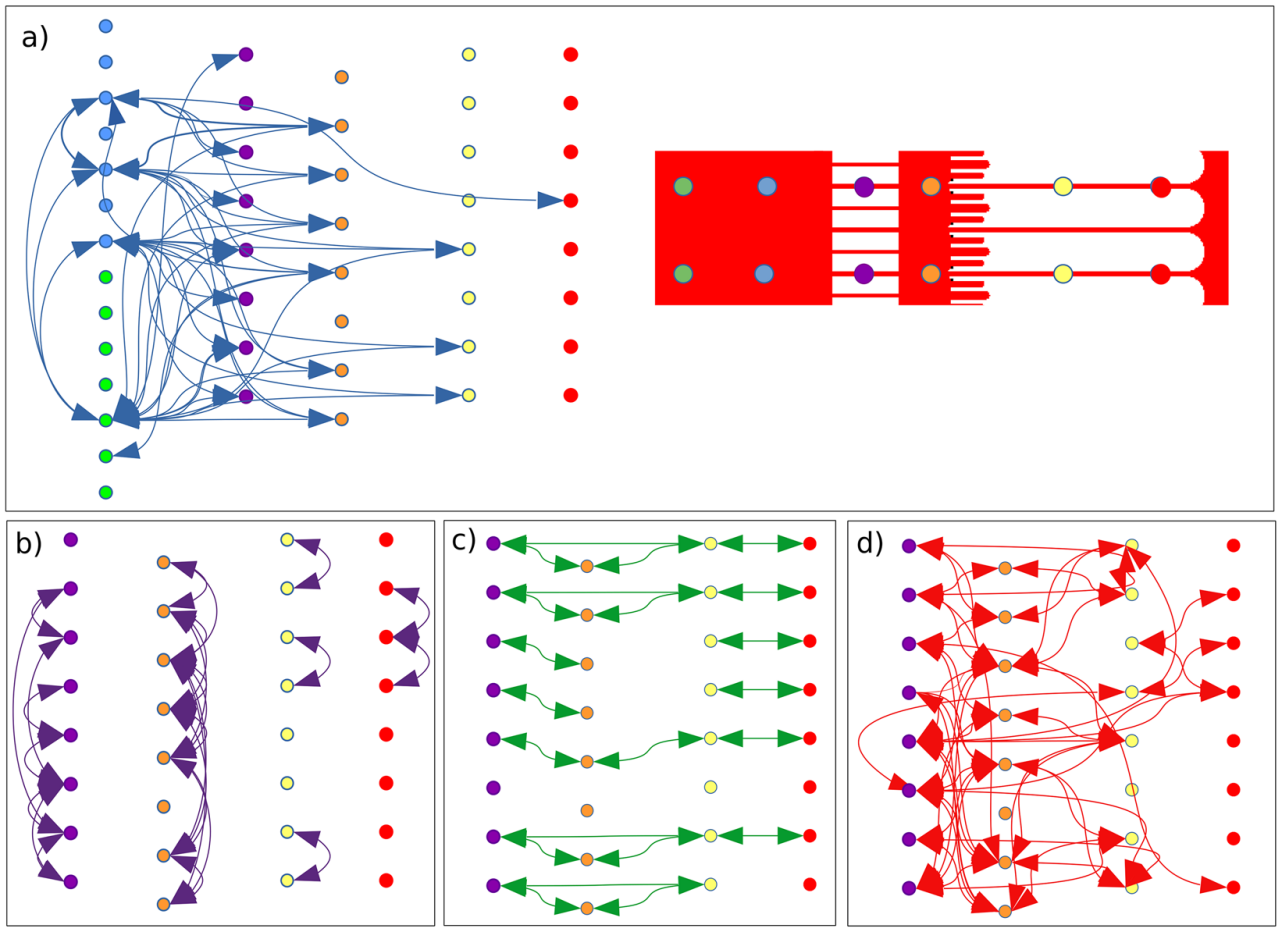
Immediate correlation of spike trains within the organized NN. Mapping of short-term correlations (signal delay is ± 2.5 ms max) extracted from the MEA recordings of 11-day-old microfluidic NNs. Arrows represent a significant correlation between the 5 ms-binned spike trains of two electrodes. The maximum delay between correlated electrodes is ± 2.5 ms. The four panels (**a**–**d**) distinguish the interactions between (**a**) somas and neurites (blue arrow) and (**b**–**d**) along neurites. (**b**) Correlation between the electrodes of the same MEA column but within different microchannels (purple arrows), showing backward and forward propagation between adjacent neurite channels or synchrony between proximal neurites resulting from the same excitation. (**c**) Correlation between electrodes of the same MEA line, thus within the same or aligned microchannels (green arrows), showing straight spike propagation; (**d**) Correlation between each electrode located within the microchannels and the synaptic chamber (red arrows), showing entangled neurite-neurite interactions. Straight correlations (green arrows, in Panel (**c**) are excluded.

The short-term cross-correlations between each microelectrode were then assessed to track signal propagation between each compartment (Fig. 6). Figure 6a first assesses the connectivity of the somatic chamber. The main feature was that there were higher correlation and synchrony levels between soma and neurite than between somas. Most of the correlations occurred with the proximal microchannels. This explains the synchrony and correlation between proximal neurites (Fig. 6b, purple column). The analysis also reveals long-range correlations with both the synaptic chamber and the axonal microchannels (orange and yellow columns). Thus, somatic signals efficiently activated the emission of spikes within distant axonal microchannels (up to a few mm).

Between different microchannels (Fig. 6b, purple arrow), the correlations appeared strongest in the synaptic chamber (n = 3.9 per electrode, orange column), where there was no physical barrier to restrict communication between neurites. Then, the correlation within different microchannels (purple, yellow, red columns) could reveal backward and forward propagation between adjacent neurite channels or synchrony resulting from the same excitation. This could stem, respectively, from closed loops of neurites (Fig. 1c) or the proximity between microchannels and the somatic or synaptic chambers. The number of these correlations was higher for proximal microchannels, both in terms of number and length of correlation, up to electrodes separated by 5 pitches (n + 5). If we consider the neural architecture as we designed it, this would suggest a higher level of connectivity for the dendrites and proximal axons (both present within the short microchannels) than for the distant axon (long microchannel). Further studies should assess this point with immunostaining to identify dendrites and axons and excitatory and inhibitory neurons, for instance. In fact, we must not neglect other possibilities, such as the impact of dendritic signals (e.g. EPSPs and IPSPs from inhibitory and excitatory neurons), which may hide activity within distant microchannels.

Figure 6c shows straight propagation along aligned microchannels (green arrow) and presumably along the same or connected neurites. Again, more signals propagated to the left than to the right side of the synaptic chamber, which agrees with the expected position of the dendrites and axons and the filtering effect of the synaptic chamber. These propagations were dominated by short-distance correlations, essentially between neighboring electrodes (n + 1 or n + 2). Long-range interactions were, however, clearly distinguished between misaligned electrodes (Fig. 6d, red arrow), with each active site being correlated on average with three distant (> n + 1) electrodes and one neighboring (n + 1) electrode. The spatial range of the correlation reached several millimeters (up to n + 6). Generally, those panels show that straight propagation involved axonal channels, while propagation between dendrites and within the synaptic chamber was more spatially distributed, which is indeed as expected for hippocampal neurons. The design architecture of the microfluidic NN is functionally relevant.

**Figure 7:**
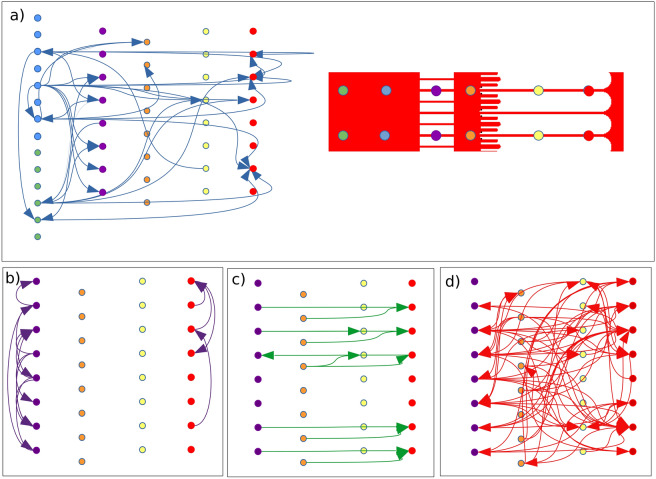
Long-term correlation of spike trains within organized NN. Mapping of delayed correlations (signal delay is ± 25 ms max) extracted from the MEA recordings of 11-day-old microfluidic NNs. Arrows represent significant correlation with a delay between − 25 ms and 25 ms between 5-ms-binned spike trains of two electrodes. Short-term correlations with a delay less than 5 ms are excluded. The four panels (**a**–**d**) distinguish the interactions between (**a**) somas and neurites (blue arrow) and (**b**–**d**) along neurites. The same representation as in Fig. 6 is used for the purple, green and red arrows.

The directionality of neural communications was then assessed by picturing the delayed cross-correlations (between 5 and 25 ms). Thus, the correlated spike trains were expected to share a similar origin. We assume that a positive delay between correlated electrodes (A and B) indicates the direction of propagation (from A to B), regardless of the propagation pathway (possibly indirect with hidden nodes). Under this assumption, most of the short-range correlations observed previously were suppressed, while long-range correlations are numerous despite the distance between electrodes and the background noise (Fig. 7).

The temporality of events was clear within aligned microchannels (Fig. 7c). Signals propagated from the short to the long microchannels toward the axons and seemed to originate from the somatic chamber (Fig. 7a). Additionally, the same somatic electrode seemed to activate several neurite channels, which could explain the correlation observed between those microchannels (Fig. 7b). Within adjacent and parallel microchannels (Fig. 7b), signals could be carried by the same neurites (in a closed loop configuration), but the delay (± 5–25 ms) suggests indirect communications, presumably by dendrites. As illustrated in Fig. 7d, communications were highly intricate between short and long channels, which confirms efficient neurite mixing within the synaptic chamber. The directionality was also mitigated, as 50% of propagations occurred in both directions for the purple and red columns (short and long microchannels). This dual directionality agrees with the emergence of both input and output nodes in the same somatic chamber (green and blue columns Fig. 7a). For that reason, we can barely distinguish backpropagation events, if any, and their impact on signal processing within such microfluidic circuits.

Interestingly, we observed only one efferent node and few (3–4) afferents (output and input nodes, respectively) for both conditions within organized and random NNs (Fig. 7a and Fig. S4, respectively). However, the number of correlated spike trains was significantly reduced in control cultures of the same age, which confirms intense activity underlying the accelerated maturation within the microfluidic environments. The microchannels are shown to enhance the detection efficiency and amplitude of recorded signals. However, high levels of activity and synchrony were also observed in the wider synaptic chamber, which excludes an isolated effect of the enhanced detection efficiency within the microchannels. Differences in encoding properties between random and organized NNs are thus demonstrated, leveraging a high level of connectivity. While somas and neurites could be isolated, this analysis indeed underlines the complexity of neural communications and the rich encoding possibility even within a basic one-node architecture.

## Conclusion

Here, microfluidic model NNs have been exploited in attempts to define neural circuits that underlie simple neuron behaviors. It is often difficult to study individual propagation or connections in detail and to rule out involvement of unidentified neurons. These limitations have been overcome by reconstruction of partial circuits that localize the neurites and by the identification of active and correlate neurons. This approach has provided opportunities to examine the emergence of spontaneous activity at both the single-cell and network level, as well as neural communications of complex neuronal circuits in a manner that is unapproachable in self-organized cell culture, by providing access to spike propagation within cell compartments; more broadly, this approach enables neural communications to be assessed general. Beyond NN structuration, an important issue was to know determine microfluidic circuits are capable of driving the functional properties of the NN. This study shows that beyond shaping the organization of the neural network, the imposition of structure increases electrical activity and axo-dendritic interactions at both the single-cell and network levels. Spiking rate, burst and correlations were reinforced over time in terms of number, intensity, and modularity greatly exceeding the electrophysiological features of standard cultures, independent of the MEA detection efficiency. This work further highlights the impact of microfluidics on cell development and its ability to shape both the architecture and activity of the neural compartment, serving as a stepping stone to the establishment of topological NNs in the laboratory.

## Materials and methods

### Spike sorting and signal analysis

All data analysis was performed with custom Python code using publicly available packages such as McsPy, Elephant, Neo and Viziphant. First, raw time series data from all electrodes of organized samples were referenced again to remove artifacts due to an important resistive path to the reference in the chamber. Electrodes in columns 1 to 4 were referenced against the reference electrode closest to the chamber containing the column 1 and 2. On the other hand, electrodes with column numbers 5 to 8 were referenced against the reference electrode closest to the chamber containing the column 7 and 8 electrodes. After this step, the data were filtered with a Bessel 4th-order bandpass filter, with cutoff frequencies of 200 Hz and 3500 Hz. Then, the time series data were converted to spike train data using a threshold function of ± 5 MAD, computed over all 50 s of the recording. The sign of the threshold was determined by assessing the first crossing between the positive and negative values, i.e. the first phase of the spike waveform. It was then used to create the spike trains by gathering the timestamps of the crossings with a post-spike dead time of 3 ms. Electrodes with a mean firing rate of 0.1 Hz across the whole recording were then labeled “active” and used in further analysis. Burst events on each electrode were defined by a succession of spikes with a maximum interspike interval of 100 ms between them.

The cross-correlation was computed for each electrode pair on spike trains binned in intervals of 5 ms; the values of both the central bin and the maximum-height bin (with its associated delay) were extracted to confirm that a significant threshold was crossed on visual inspection. Then, using those values, graphical representations of neural communications were constructed. The correlation matrices were computed directly from the binned spike trains using the Elephant package.

### Sample preparation

The PDMS chips were manufactured by molding the negative image of on-wafer photoresist structures. Briefly, the chambers and microchannels were lithographed in two steps using different thicknesses of MR-DWL (40 µm and 5 µm, respectively; Microresist Technology GmbH) and backside alignment. Before Sylgard 184 PDMS (Dow Corning) was poured, the resulting mold was hard-baked on a hot plate at 180 °C for 30 min. We used a curing duration of 2 h at 80 °C to ensure that the polymerization was complete and that the residues would not be able to affect the culture. Openings were punched for fluidic access, and then the chips were sterilized by placing them in 70% EtOH/H_2_O and sonicating them. Commercial MEA chips (60MEA200/from Multichannel Systems) were placed inside a plasma chamber to clean them and make them hydrophilic (100% O_2_, 120 mTorr, 120 s, March PX500), after which they were immediately sterilized with 70% EtOH. Then, the organized samples were assembled under an inverted microscope by using the ethanol to slide the PDMS chip channels above the electrodes. All samples were left to dry in a biosafety cabinet, exposed to UV light and rinsed three times with sterile DI water.

### Cell culture

E16 mouse embryos were used to provide primary hippocampal neurons according to previously released protocols (NMRI mouse Janvier Lab)^51^. Briefly, a suspension of 10 million cells/mL in MEM containing 10% horse serum, 0.5% penicillin/streptomycin and 1% glutamine (Thermo Fisher Scientific Inc.) was made. For the organized samples, in each well, 10 µL of the solution was injected away from the chamber entrance, starting at one side, waiting 1 min for the cells to settle and filling the other side afterward. For the random samples, the suspension was diluted by a factor of 50, and new cultures were seeded with 1 mL of solution. All samples were left for 10 min to allow cells to adhere. The samples were placed inside an incubator with a 5% CO_2_ atmosphere. Two hours later, the wells of the PDMS chips were filled with 100 µL fresh media (glia-conditioned Neurobasal). The media were changed every 48 h, compensating for evaporation. No live animals or humans were used in the study.

### Immunostaining

Cells were fixed in 3.7% paraformaldehyde (diluted in PBS) for 10 min, permeabilized with 0.1% Triton X–100 for 5 min and blocked in PBS-2% bovine serum albumin. After being fixed, cells were incubated with DAPI (Invitrogen) to mark the nuclei, and pairs of primary and secondary antibodies were chosen to locate the neurites (anti-YL1/2) and the synapses (anti-synapsin); these antibodies were diluted in PBS containing 10% bovine serum albumin before being applied. Fluorescence micrographs were collected using either an Olympus BX51 or a Zeiss AxioImager M2 microscope.

## Supplementary Information

Supplementary Figures.

## Data Availability

The datasets used and/or analyzed during the current study are available from the corresponding author on reasonable request.
