# Supplementary material for: Portrait of intense communications within microfluidic neural networks
Source: Sci Rep. 2023 Jul 29;13:12306. doi: 10.1038/s41598-023-39477-9 (PMC10387102; doi:10.1038/s41598-023-39477-9)
Supplement: Supplementary file 1 — Supplementary Figures. [file 41598_2023_39477_MOESM1_ESM.pdf]

Portrait of intense communications within microfluidic neural networks

Victor Dupuit,[1] Anne Briançon-Marjollet,[2] Cécile Delacour[1*]

1 Institut Néel, University Grenoble Alpes, CNRS, Grenoble INP, 38000 Grenoble, France

2 University Grenoble Alpes, HP2 Laboratory, Institut National de la Santé et de la Recherche Médicale U1300, Grenoble, France

Supplementary figures from S1 to S4

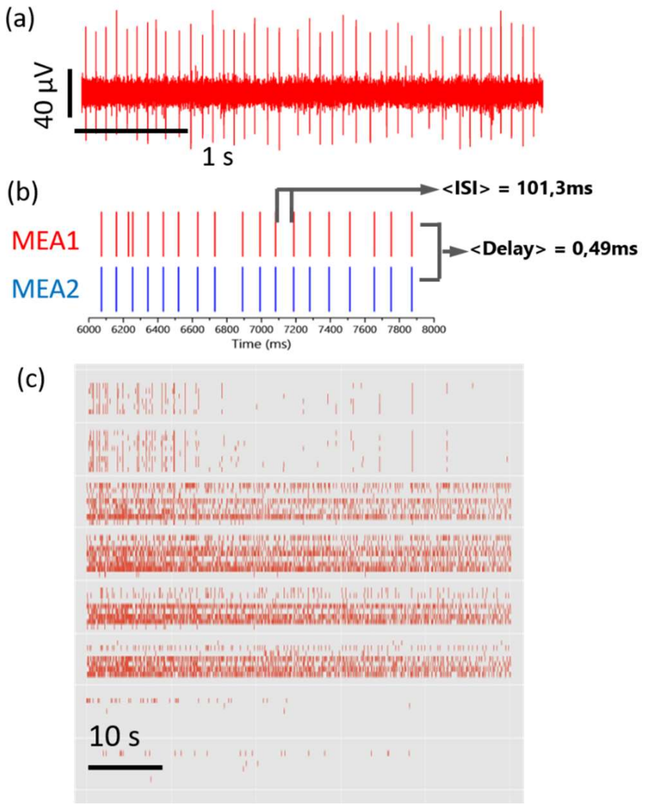

Figure S1. Dual-active compartments microfluidic NN. (a-c) Multisite recording with MEA array assembled with a microfluidic neural network as shown in figure 1. (a) Typical voltage-time trace recorded with a microelectrode and (b) zoom in the raster plot of two successive microelectrodes within a same microchannel (extracted from panel c). The delay between each spike (+0.49 ms) is as expected for spike propagating along an axonal microchannel located between two somatic chambers. (c) Raster plots of all the 60 microelectrodes aligned along the fluidic microchannels and with two filled somatic chambers. Neurons exhibit activity in the two somatic chambers. Day-in-vitro 5.

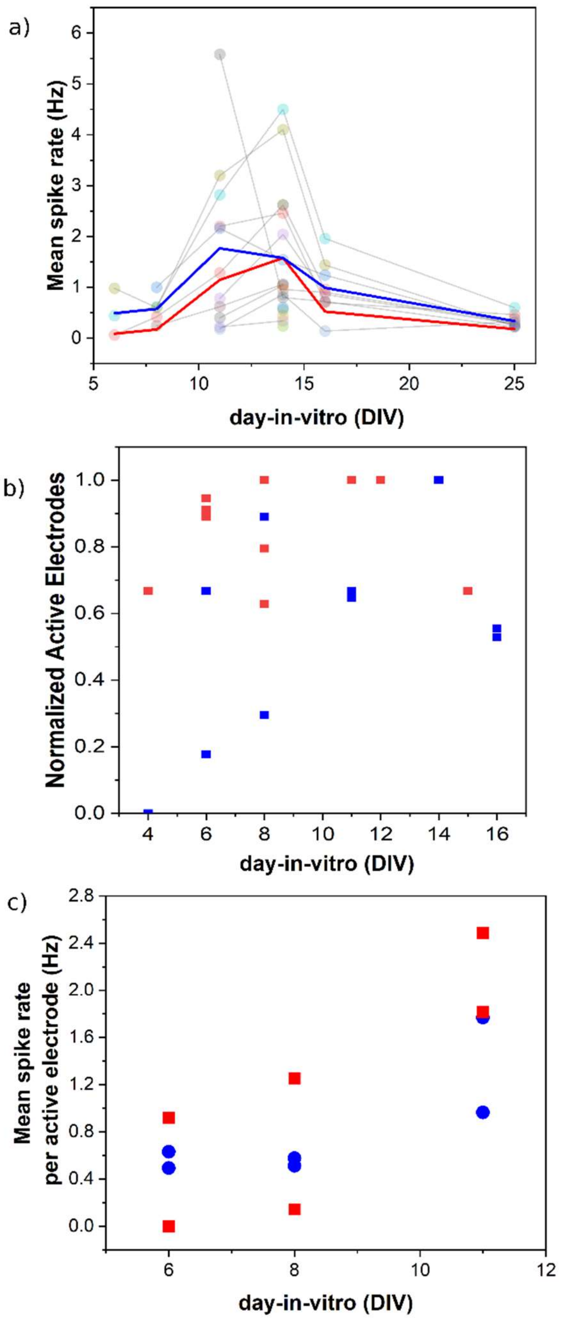

Figure S2. Spontaneous activity of random and organized NNs across the culture time. (a) Evolution of the mean spike rate on active electrodes (min 0.1 Hz mean firing rate) versus the DIV of a typical random sample. The blue line and the red line represent the mean by the maximum number of active electrodes on each day and by the maximum number of active electrodes achieved independently of the day. (b) Scatter plot of the normalized active electrodes versus the day-in-vitro DIV. Normalization is performed by dividing the number of active electrodes by the maximum number of active electrodes recorded for the sample. Blue are for random samples and red for the organized ones. (c) Mean spike rate per active electrodes on random and somatic regions from the control (blue) and organized (red) samples resp. versus the DIV.

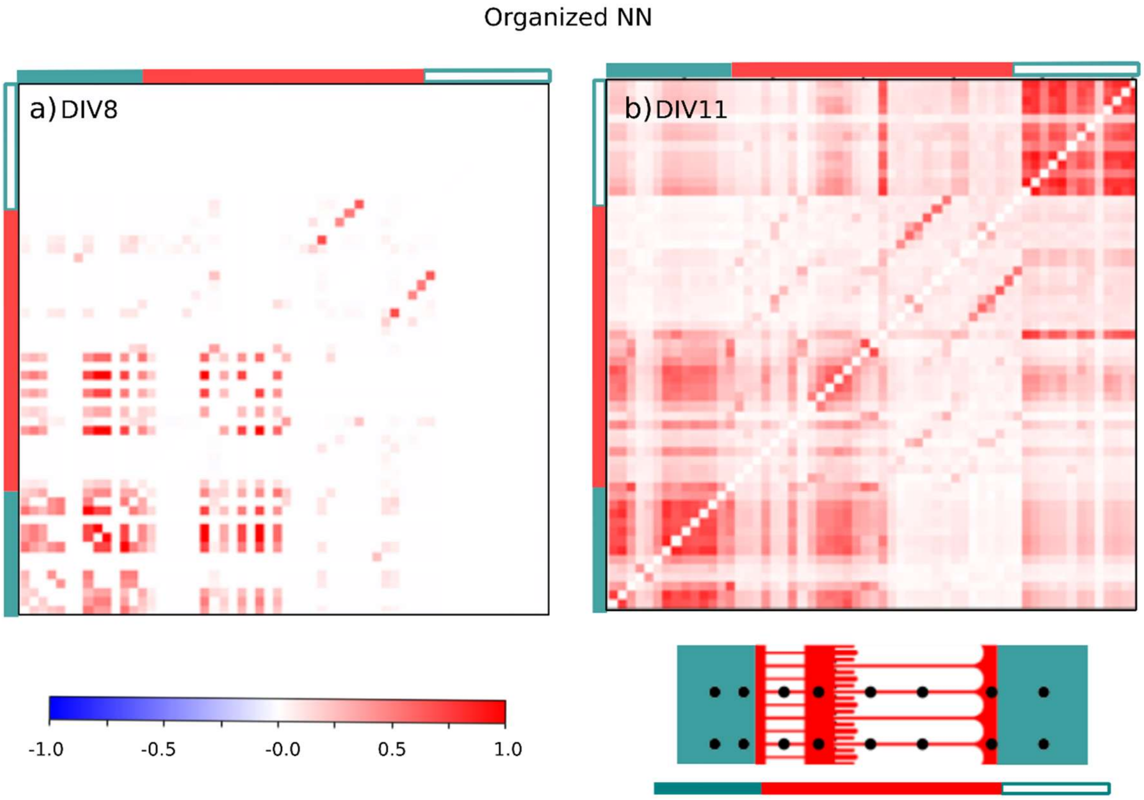

Figure S3. Mid-term evolution of the correlation matrixes between the 60 electrodes of an organized neural networks at DIV8 (left) and DIV11 (right), showing the emergence of correlation between the two somatic chambers confirming the establishment of synaptic contact between the two separated populations.

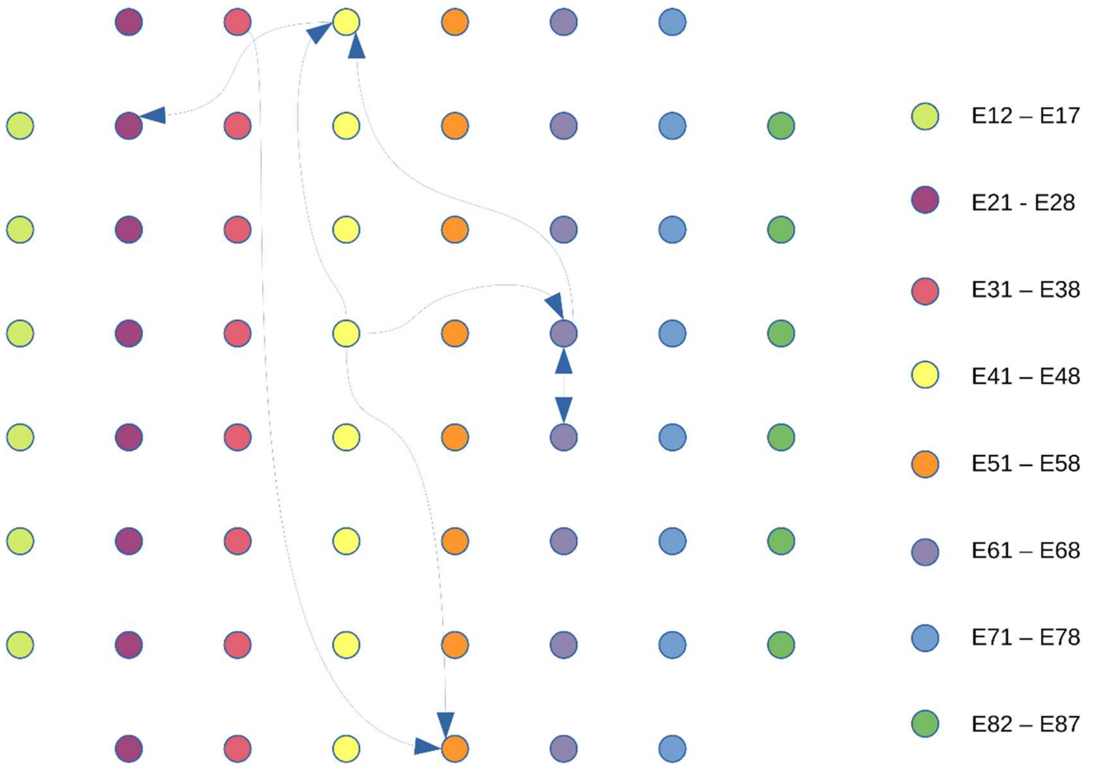

Figure S4. Landscape of neural communications within random NN. Mapping of correlated signals between electrodes in a DIV11 random sample recording. Simple arrows represent a significant correlation with a delay between -25ms and 25ms, excluding delays inferior to 5ms, between 5ms-binned spike trains of two electrodes. Double arrows represent a significant correlation at the central bin between the 5ms-binned spike trains of two electrodes.
